# S143. NEURAL CORRELATES OF INTENTION AND BELIEF INFERENCE RELATIVE TO EMOTION ATTRIBUTION TO OTHERS IN SCHIZOPHRENIA AND PSYCHOSIS PRONENESS: ACTIVATION LIKELIHOOD ESTIMATION META-ANALYSIS

**DOI:** 10.1093/schbul/sby018.930

**Published:** 2018-04-01

**Authors:** Ksenija Vucurovic, Stéphanie Caillies, Arthur Kaladjian

**Affiliations:** 1 Laboratory Cognition, Health and Society, University of Reims Champagne Ardenne; 2 Laboratory Cognition, Health and Society, University of Reims Champagne Ardenne, University Hospital in Reims

## Abstract

**Background:**

Social cognition can be briefly defined as the ability to interact with and understand others. It involves several cognitive processes that are considered as critical for an adapted social functioning. In patients with schizophrenia (SCZ) and subjects prone to psychosis (PP), a number of studies have revealed impairments in the abilities to infer beliefs, intentions or emotions of others. Cognitive tasks specifically addressing these abilities have also revealed abnormal neural processing in these subjects. However, these studies have not yet been compared in order to identify shared or distinct functional brain networks underlying these processes in the two subject groups.

Therefore, we aimed to determine whether the neurofunctional correlates of intention/belief attribution are distinct from those of emotional inference in SCZ and PP compared to healthy controls (HC). We further attempted to identify neuroimaging markers of psychosis endophenotype in mentalizing tasks. Finally, we examined shared and distinct brain regions involved in intention/belief attribution relative to emotional inference in SCZ.

**Methods:**

Using a neural coordinate-based Activation Likelihood Estimation (ALE) meta-analysis, we investigated differences in activation patterns between intention/belief and emotion attributions to others in SCZ and PP relative to HC.

**Results:**

We selected 33 studies after a systematic review of the literature. Inferring intentions/beliefs in SCZ patients correlated with decreased functional activation in the medial prefrontal cortex (mPFC) and left posterior temporoparietal junction (TPJ). In PP subjects, precuneus, posterior cingulate gyrus, middle and superior temporal gyri displayed additional under-activation pattern, while posterior cingulate, right TPJ, left lateral PFC and insula were over-activated. In patients with SCZ thalamus and striatum, right dorsolateral PFC, right insula, and right transverse temporal gyrus were under-activated during emotion attribution to others, while left ventrolateral PFC, left insula, right lingual gyrus and areas in the cerebellum were over-activated. Finally, in PP subjects, right TPJ was under-activated while left parahippocampal, middle and superior temporal gyri, were over-activated during affective mentalizing. Conjunction analyses demonstrated under-activation in left rostral mPFC and left fusiform gyri in both SCZ and PP relative to HC during intention/belief inference tasks.

**Discussion:**

Our results suggest abnormal neural functioning in fast emotional appraisal and subsequent cognitive modulation during emotion perspective taking in SCZ. In PP, abnormal activation was observed only in cortical regions well known as recruited in emotional top-down regulation. When there is no emotional content in perspective taking like in intention/belief attribution to others, two core regions appeared as under-activated in SCZ and PP, namely left rostral portion of mPFC and, to a lesser extent, the left fusiform gyrus, suggesting that these two regions play a role in top-down modulation of cognitive mentalizing and could be neuroimaging markers of psychosis endophenotype. Thus, abnormal functioning in these specific brain areas could be a valuable predictor for developing schizophrenia in at-risk subjects. Moreover, these brain regions could be targeted by non-invasive neuromodulation techniques in order to restore cognitive function.

